# Successful Retrieval of a Fractured Coronary Angiography Catheter Using Snare Technique: A First Case Report From Afghanistan

**DOI:** 10.1002/ccr3.70785

**Published:** 2025-08-27

**Authors:** Abdul Qadir Nawabi, Christian Sanchez, Aya Ziq, Mukhtaar Ahmed, Zarina Razaie, Noor Ahmed Jamal, Ahmad Jamshed Rezaie

**Affiliations:** ^1^ Mellat Medical Institute Kabul Afghanistan; ^2^ Dr. Kiran C. Patel College of Osteopathic Medicine Nova Southeastern University Fort Lauderdale Florida USA

**Keywords:** cardiology, cardiovascular disorders, coronary angiography, foreign bodies, imaging

## Abstract

This case highlights the significance of adaptability and multidisciplinary teamwork when managing vascular complications. It highlights how basic tools, such as the snare device, can effectively retrieve fractured coronary catheters in resource‐limited settings, potentially guiding future practices toward practical, cost‐effective, and innovative solutions in similar clinical scenarios.

## Graphical abstract

Fractured catheter fragment lodged in the internal iliac artery before retrieval with a snare.

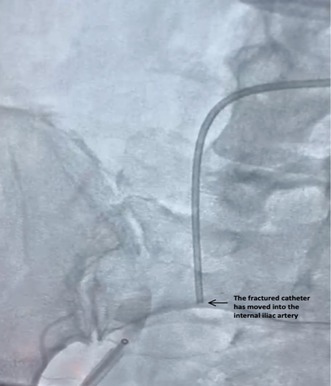

## 1 Introduction

Coronary angiography is an important diagnostic and interventional tool for evaluating coronary artery disease (CAD). While generally safe, complications can arise, including vascular injury, arrhythmias, and in rare cases, catheter fractures [1]. Tortuosity in the vasculature, particularly in arteries such as the iliac or aorta, poses more challenges, as it increases the mechanical stress on catheters, potentially leading to kinking or breakage [2, 3, 4]. Literature has documented catheter‐related complications, but catheter fractures are particularly uncommon and have been linked to adverse outcomes like embolization or arrhythmias [5, 6]. The management of such complications usually involves advanced retrieval techniques or, in severe cases, more invasive surgical intervention.

However, most available literature focuses on managing these complications with advanced equipment, overlooking practical challenges encountered in resource‐limited environments. Specifically, there is limited information on the impact of severe vascular tortuosity on catheter fractures and how fragment location, particularly in high‐risk areas like the left ventricular apex, affects complication severity and retrieval difficulty [7]. Timely retrieval remains crucial to prevent severe complications such as thrombosis, ischemia, infection, or death [8].

This report highlights a unique case of catheter fracture caused by significant tortuosity of the iliac arteries and aorta, resulting in the catheter tip lodging in the left ventricular apex. We emphasize the critical importance of adaptability, demonstrating how effective utilization of basic retrieval tools, like a snare device, can safely manage complex catheter complications in resource‐constrained settings.

## 2 Case History/Examination

A 57‐year‐old female with a history of hypertension, a left ventricular ejection fraction (LVEF) of 40%, and dilated left heart chambers presented with complaints of dyspnea on exertion. She had no significant history of other cardiovascular risk factors. A coronary angiography was indicated to evaluate for coronary artery disease. The procedure was started through the conventional femoral approach, and the left main coronary artery was successfully cannulated using a Judkin left 3.5 catheter, 6 French (F).

**FIGURE 1:**
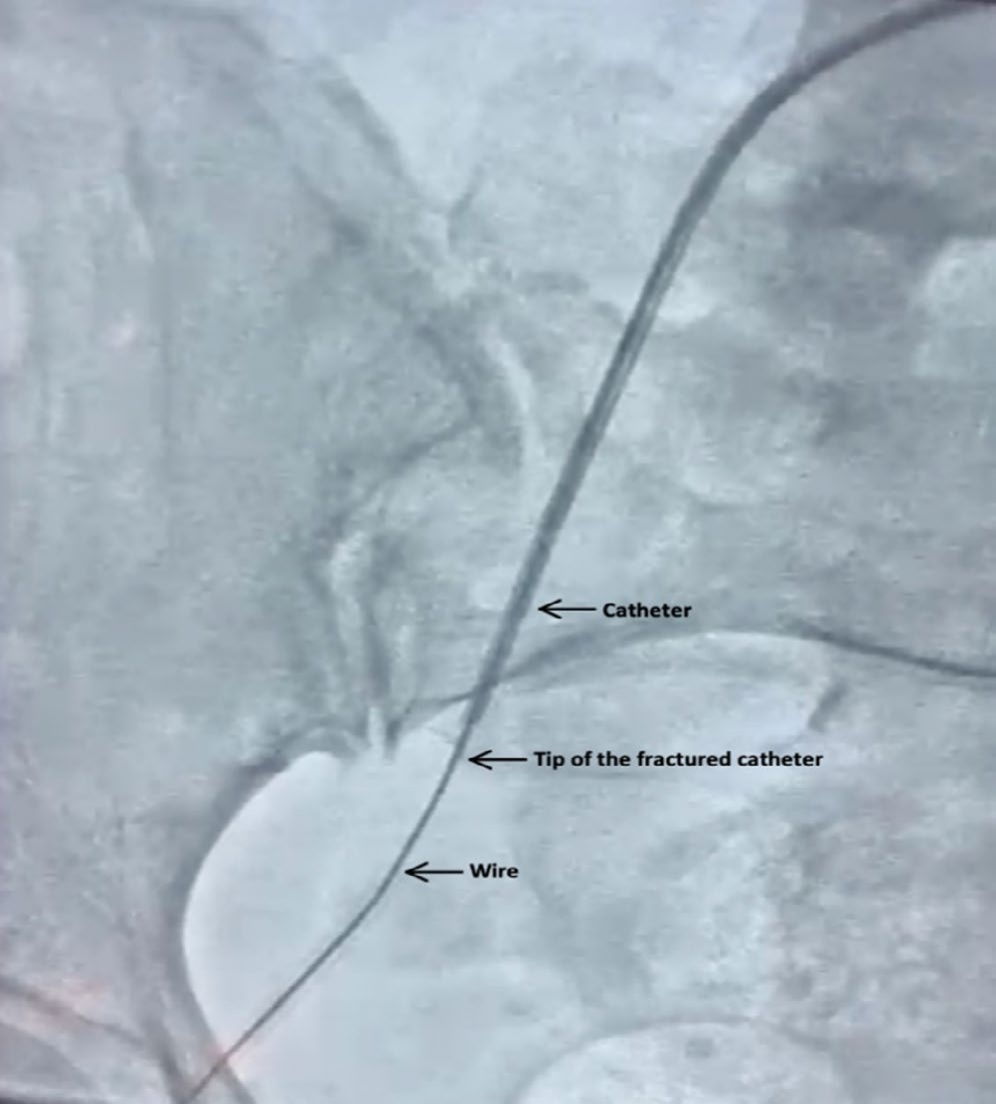
Initial imaging of fractured catheter. Angiogram showing the fractured catheter segment lodged in the left external iliac artery, with guidewire and catheter pathway visible.

**FIGURE 2:**
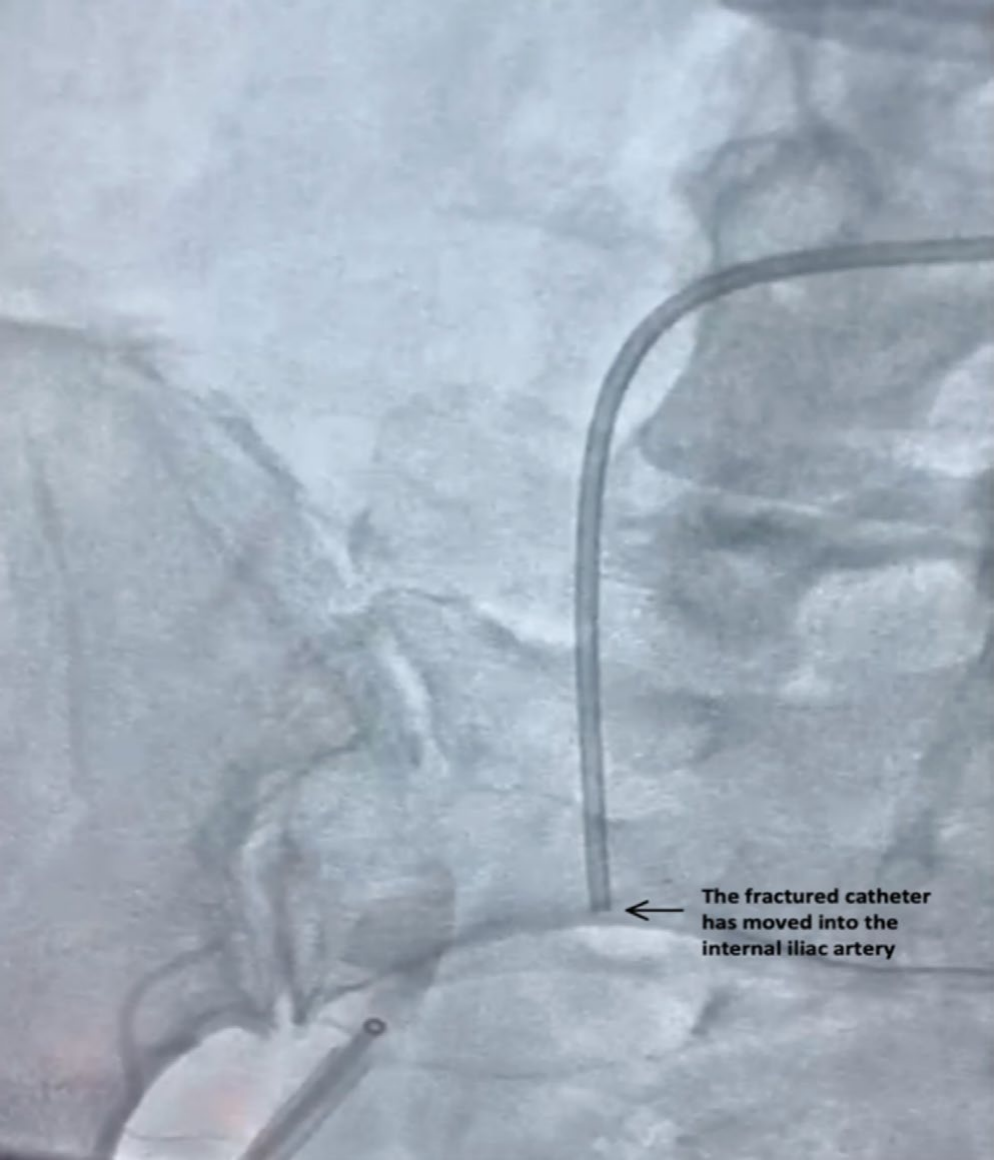
Fractured catheter migration. The catheter fragment is visualized within the right internal iliac artery, illustrating its migration and procedural complexity.

**FIGURE 3:**
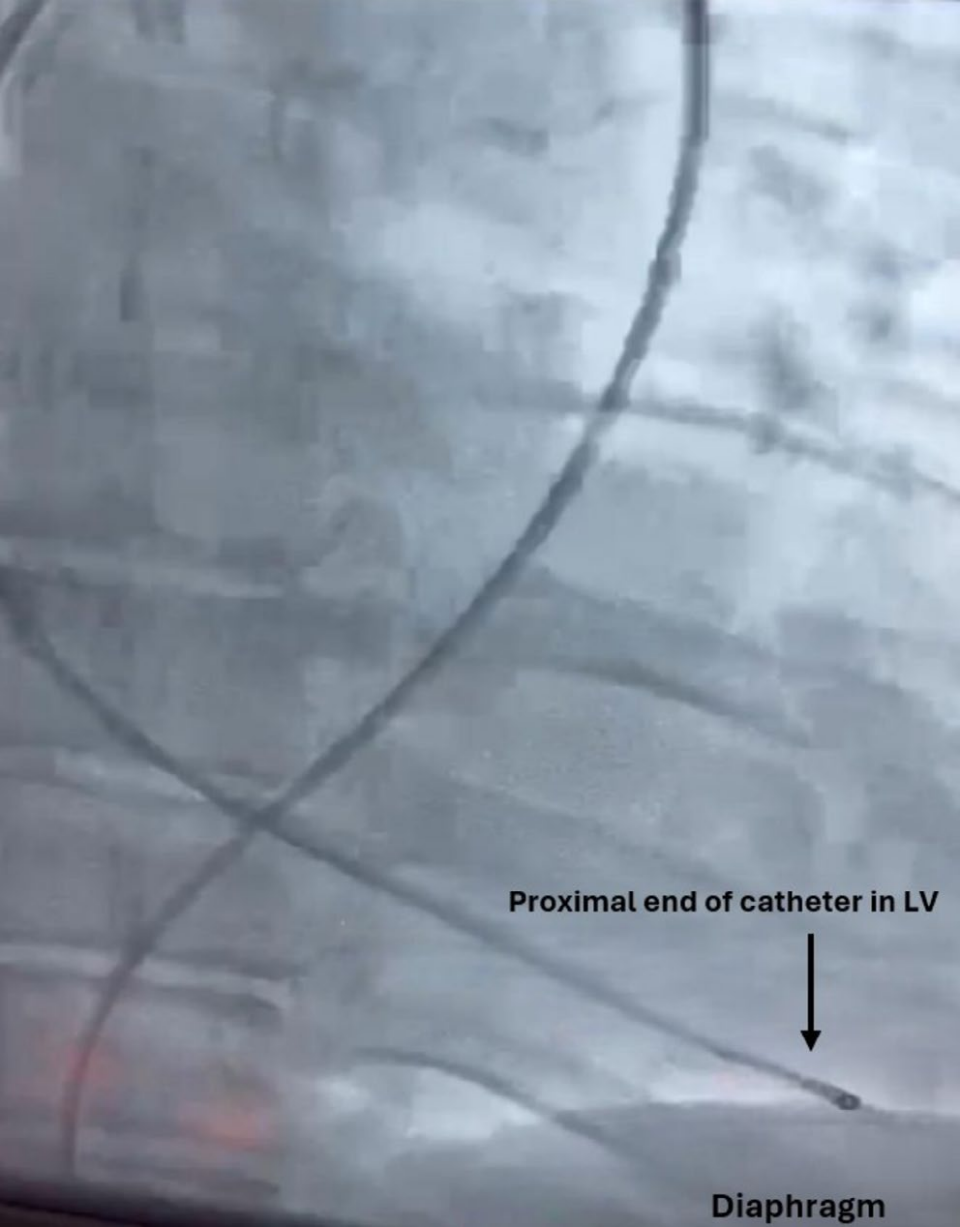
Catheter and wire orientation. Imaging reveals the proximal catheter fragment embedded in the left ventricular apex, highlighting arrhythmia risk and retrieval difficulty.

**FIGURE 4:**
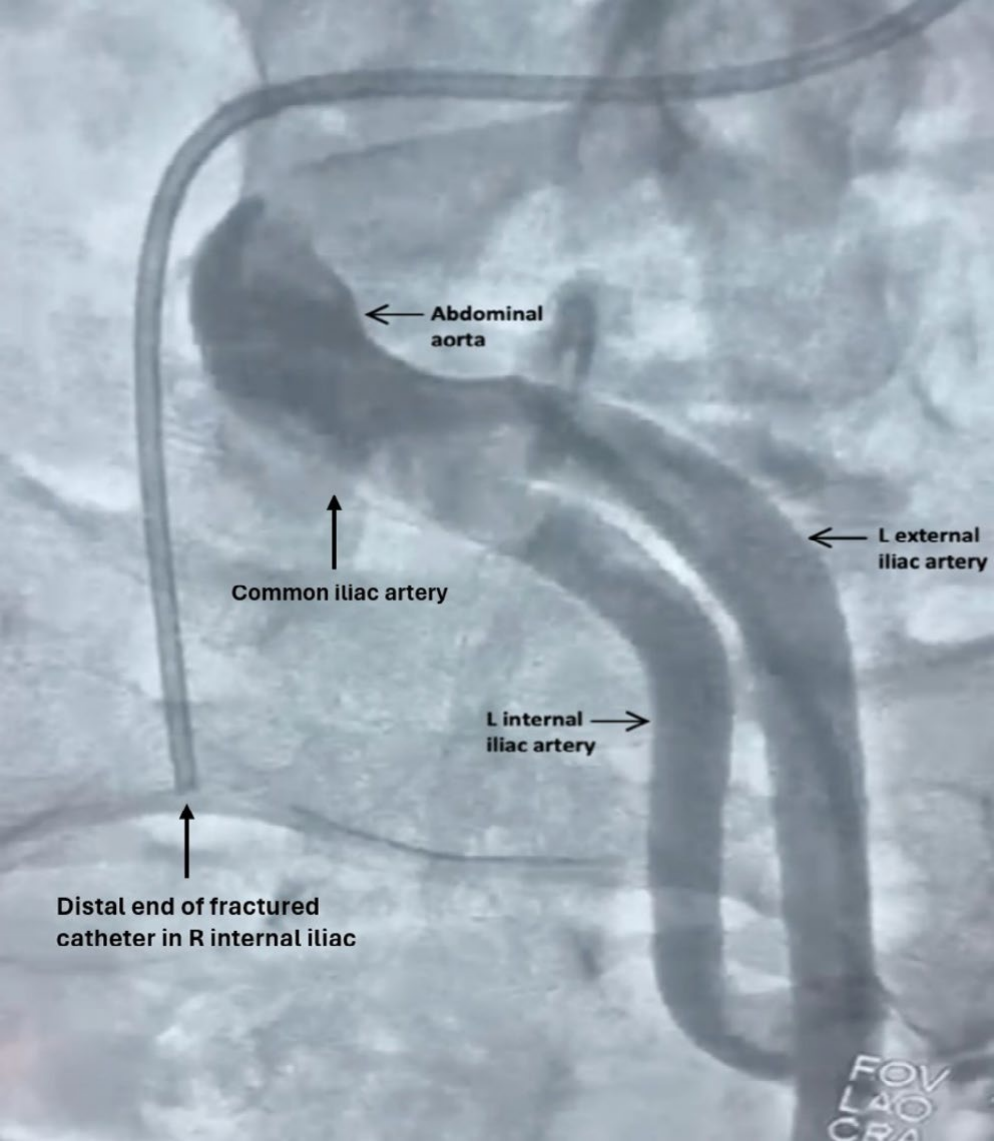
Abdominal aorta and iliac artery branching. Angiographic view of the abdominal aorta and iliac branches, contextualizing the catheter's path relative to major vascular structures.

During right coronary angiography, the tortuosity of the patient's external iliac artery, abdominal aorta, and thoracic aorta created excessive traction on the catheter, causing it to kink and fracture (Figure 1). The distal portion of the catheter migrated into the right internal iliac artery while the tip became lodged in the left ventricular apex, causing multiple premature ventricular contractions (PVCs) (Figures 2 and 3). Further angiographic visualization (Figure 4) of the abdominal aorta, left external iliac artery, left internal iliac artery, and common iliac artery provides context for the location of the catheter fracture, showing its proximity to major vessels and emphasizing the importance of precision in navigating these structures. The patient remained hemodynamically stable throughout the procedure.

## 3 Differential Diagnosis, Investigations, and Treatment

### 3.1 Angiography of the Right Iliac Artery

To further assess the anatomy and secure vascular access, a 7 French sheath was inserted from the left femoral artery, allowing access to the right iliac system for retrieval of the fractured catheter. An angiogram of the right iliac artery was performed to assess the condition of the vasculature and identify any potential complications from the catheter fracture. The angiogram revealed a dissection of the external iliac artery. Despite the dissection, antegrade blood flow was observed in the right common and external iliac arteries, which was a reassuring finding.

### 3.2 Vascular Support for Retrieval

Once the dissection was identified on the right side and antegrade flow was confirmed, a hydrophilic diagnostic wire was carefully advanced into the right internal iliac artery from the left femoral artery. This wire was then exchanged for an extra‐stiff wire, providing better stability and support during the retrieval procedure. A 6 French multipurpose catheter was then inserted over the extra‐stiff wire and positioned in the internal iliac artery to facilitate further maneuvers. An angiogram was performed using the 6 French catheter to locate the exact position of the fractured catheter and confirm its migration to the iliac system. The angiogram also allowed for the visualization of the catheter's relationship to the surrounding vasculature.

### 3.3 Snare Technique and Initial Attempt

The first retrieval attempt was performed using a Goose neck EV3 snare (Covidien/Medtronic) with a 55 mm loop. This device was introduced via the left femoral sheath over the wire and positioned near the fractured catheter tip. Despite several attempts, the larger loop (55 mm) was unable to secure the distal end of the catheter.

### 3.4 Second Snare Attempt With a Smaller Loop

A second attempt was made using a smaller Goose neck EV3 snare with a 25 mm loop. This smaller snare was successfully maneuvered and locked around the distal end of the fractured catheter within the iliac artery. Once the catheter was captured, the snare was carefully manipulated to avoid dissection or injury to the smaller caliber arteries.

### 3.5 Controlled Retrieval From the Common Iliac to the Abdominal Aorta

To minimize the risk of vascular injury, the snare was initially unlocked, allowing gradual withdrawal of the catheter from the right common iliac artery back into the larger‐caliber abdominal aorta. The catheter was pulled slowly and under fluoroscopic guidance, ensuring that no additional stress was placed on the external iliac artery, which had already shown signs of dissection. Once the catheter was in the abdominal aorta, the snare was locked again to securely hold the fractured catheter.

### 3.6 Sheath Exchange and Final Catheter Removal

**FIGURE 5:**
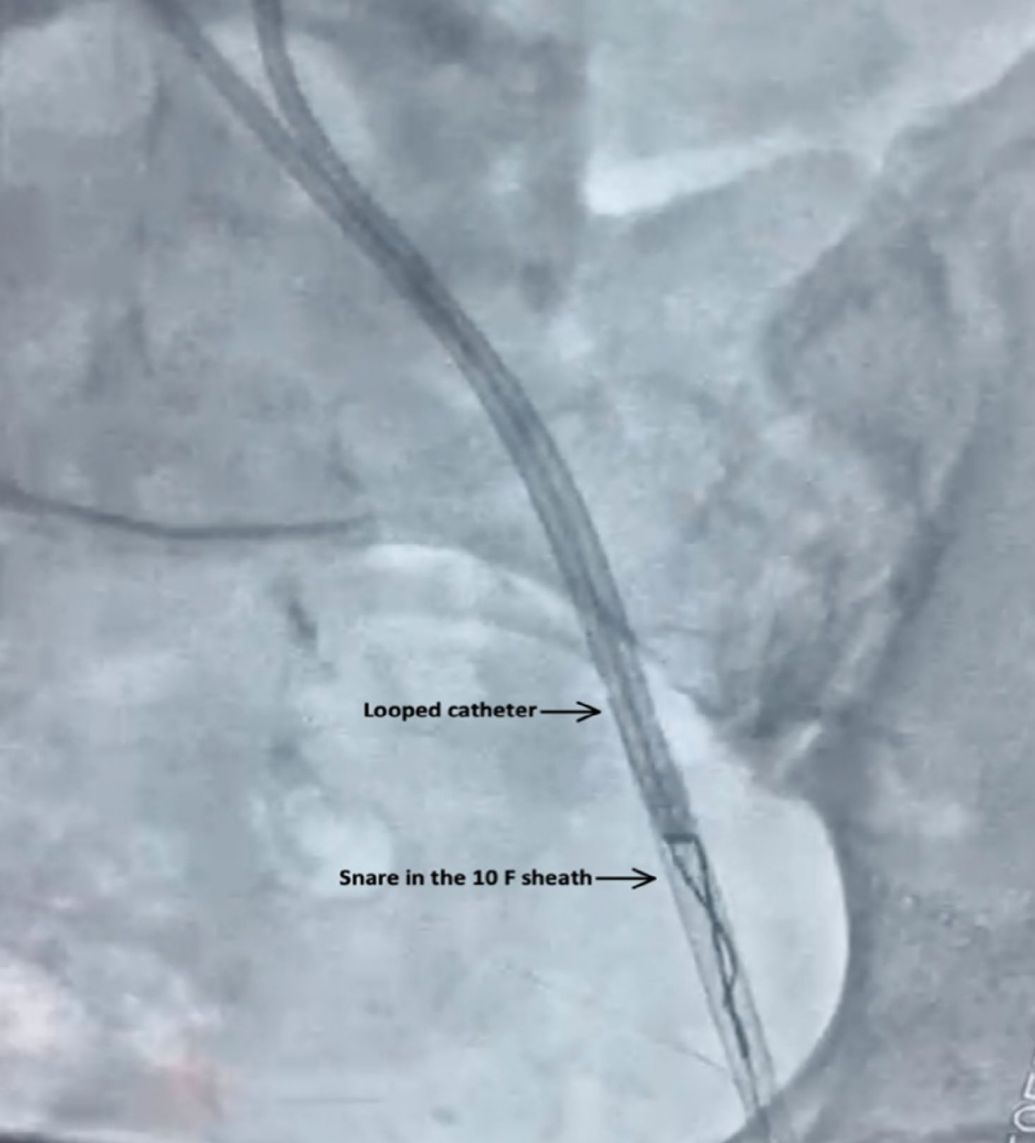
Snaring of looped catheter. The looped catheter fragment is secured by a Goose neck snare within an 11F sheath, demonstrating minimally invasive retrieval.

**FIGURE 6:**
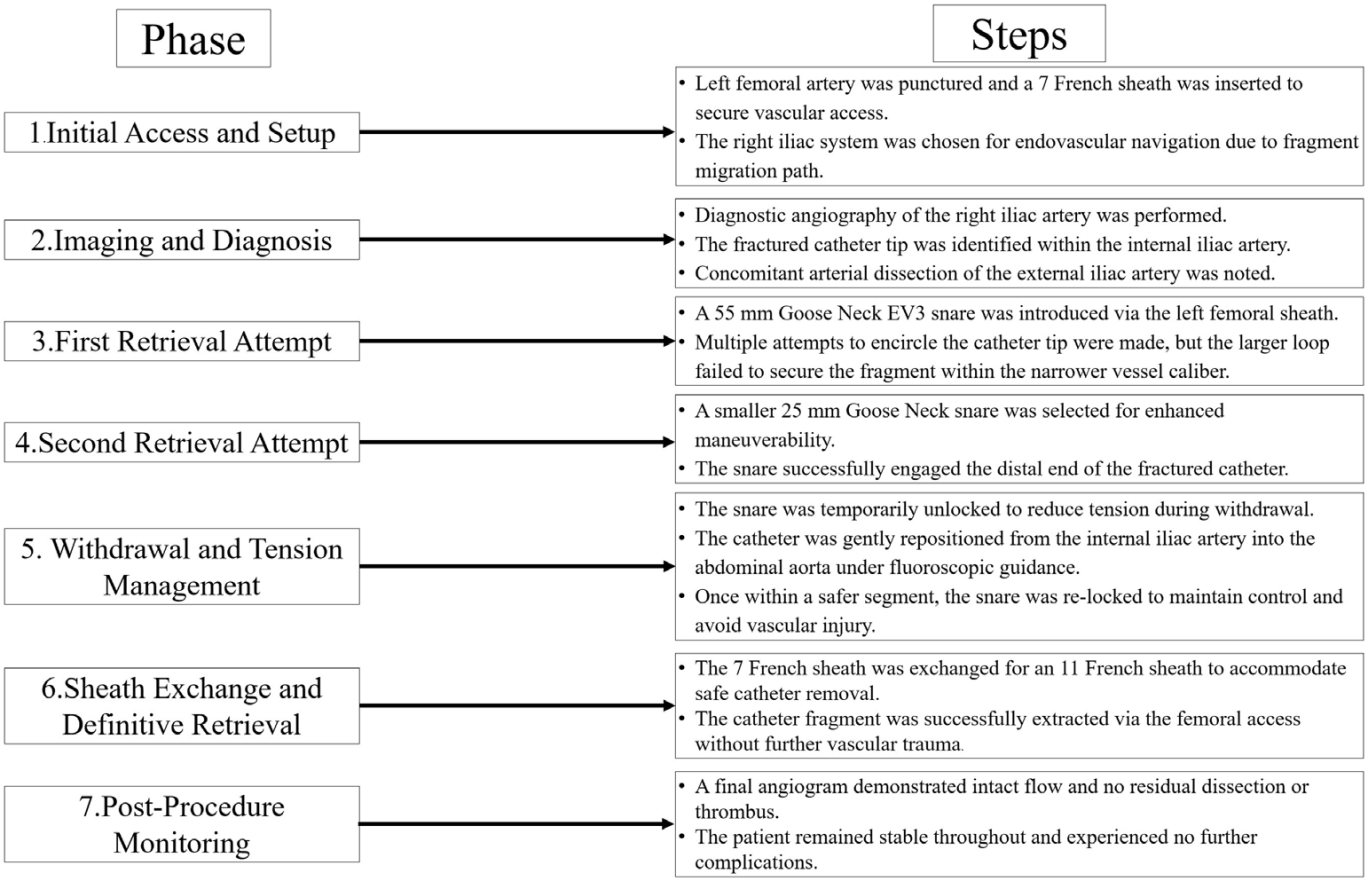
Procedural flowchart. Stepwise schematic of fractured coronary catheter retrieval using snare technique.

At this point, the right femoral sheath was exchanged once again, this time from a 7 French sheath to an 11 French sheath to allow for safe retrieval of the kinked and fractured catheter (Figure 5). The fractured catheter was then pulled down through the common iliac artery and safely retrieved from the left femoral artery. Care was taken to ensure the catheter was not causing additional damage as it was removed. The procedural flowchart (Figure 6) summarizes each step of the retrieval process, from vascular access and snare deployment to sheath upsizing and final extraction.

### 3.7 Post‐Retrieval Angiogram and Monitoring

**FIGURE 7:**
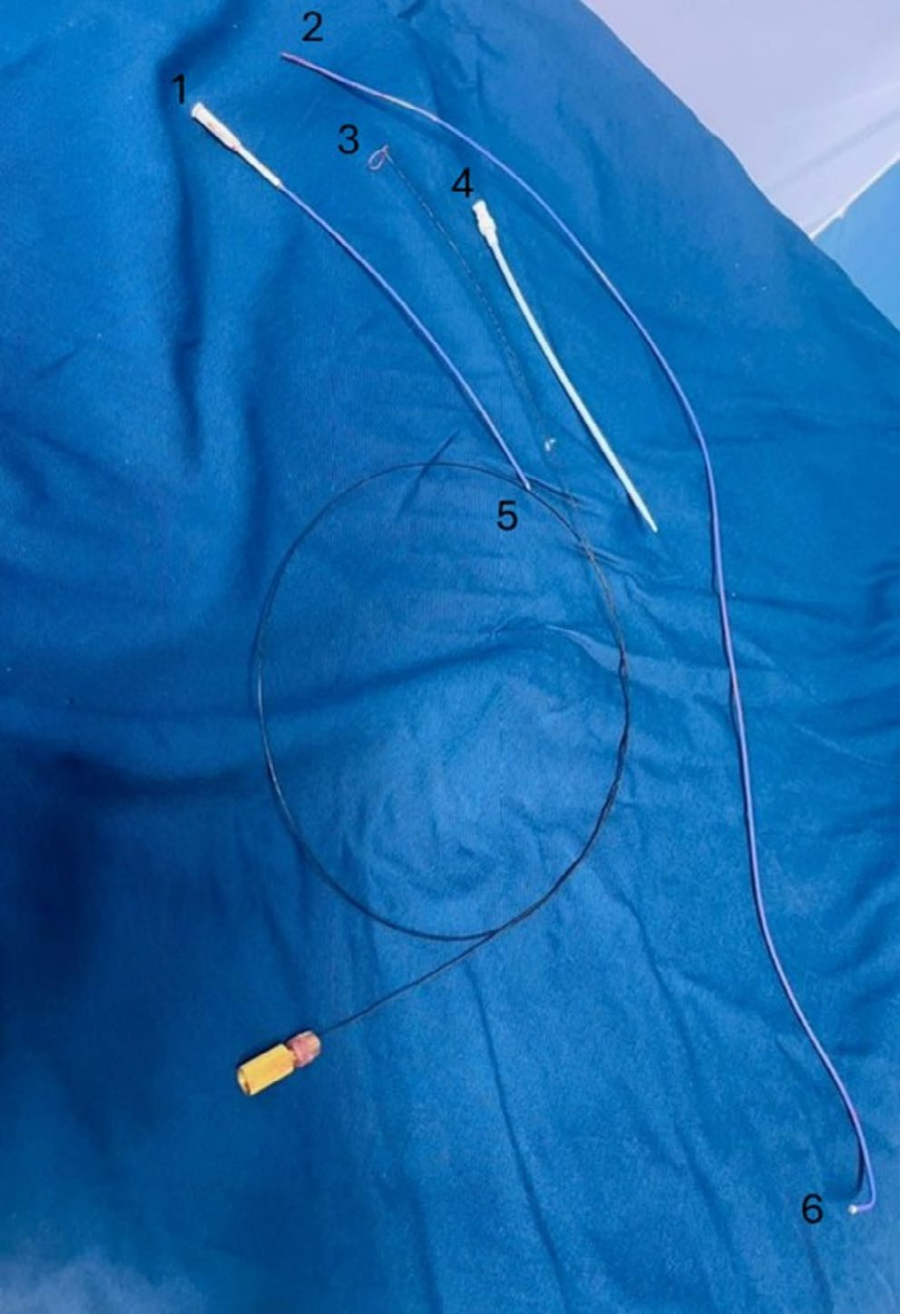
Retrieved medical equipment. This final image shows the (1) distal part of the fractured catheter, (2) distal end of the proximal fractured catheter, (3) SNARE, (4) 11 French sheath, (5) proximal end of the distal fractured catheter, and (6) proximal end of the catheter previously in the LV.

After the successful retrieval of the catheter, a post‐retrieval angiogram was performed to ensure the integrity of the vascular structures. The angiogram confirmed that there was no additional injury, dissection, or compromised blood flow in the abdominal aorta, iliac arteries, or femoral arteries. The procedure was concluded with no immediate complications. The patient was closely monitored for any post‐procedural issues, including potential vascular injury, hemodynamic instability, or recurrence of arrhythmias. Despite the initial PVCs, the patient remained stable throughout the recovery period. A cardiothoracic surgeon was on standby during the entire procedure in case of any surgical emergencies, but no surgical intervention was required. A comprehensive visual overview of the medical equipment utilized during the retrieval process highlights the complexity and success of the procedure (Figure 7).

## 4 Outcome and Follow‐Up

The patient was closely monitored post‐procedure, showing no immediate complications. Follow‐up evaluations were conducted at 1 week and 1 month after the procedure to assess for any late complications. During these follow‐ups, the patient reported no discomfort or issues, and the examination confirmed that there were no vascular complications or disruptions in blood flow, particularly in the right lower limb, despite the previous dissection noted during the catheter fracture. The patient's recovery was uneventful, and no further interventions were required.

## 5 Discussion

This case represents a rare and challenging situation of catheter fracture during a coronary angiography procedure, intensified by the patient's severe vascular tortuosity and limited resources in a developing country. Catheter fractures are uncommon complications (< 1%) that carry significant complications including embolization, arrhythmias, vascular injury, thromboembolic events, and death [9]. In this patient, extensive tortuosity in the external iliac artery, abdominal aorta, and thoracic aorta created mechanical stress that led to catheter kinking and fracture. Fragment migration to the left ventricular apex and right internal iliac artery further complicated retrieval and heightened clinical risk.

The case highlights the importance of multidisciplinary collaboration and clinical adaptability in overcoming procedural challenges in resource‐limited settings. The use of a Goose neck EV3 snare facilitated controlled, minimally invasive retrieval, avoiding the need for open surgery. Previous reports have affirmed the utility of snare devices in similar contexts [10]. While alternative techniques—as well as balloon‐assisted retrieval, forceps extraction, or surgical removal—are available, they often require more advanced infrastructure and carry higher procedural complexity.

Severe vascular tortuosity further complicates both catheter navigation and retrieval, as mechanical strain increases susceptibility to device failure. Studies have linked tortuous arterial anatomy with elevated catheter‐related complications, emphasizing the value of pre‐procedural imaging and meticulous technique [11, 12]. In these cases, thorough anatomical assessment and strategic catheter manipulation were crucial preventive measures. In other cases that reported catheter migration into the left ventricular apex, which is a high‐risk area known for arrhythmogenic potential, some patients developed transient premature ventricular contractions [13]. Prompt endovascular retrieval mitigated the risk of sustained arrhythmias. Literature consistently stresses the importance of timely intervention to prevent embolic, ischemic, infectious, or fatal sequelae, as evidenced by case series linking delayed retrieval to ventricular fibrillation and hemodynamic collapse [14].

A critical review of the literature reveals a diverse range of retrieval strategies tailored to device type, anatomical location, and resource availability [15, 16, 17, 18, 19, 20, 21, 22] (Table 1). Our case, which involved dual‐site retrieval from both the left ventricular apex and the right internal iliac artery, stands out for its successful resolution using only basic tools in a low‐resource setting. In contrast, other published cases have predominantly occurred in high‐resource environments and utilized advanced adjuncts such as sonography‐guided snaring in radiolucent catheter fractures [19], dual arterial access and balloon fixation for stent entrapment [20], or stent retrievers with aspiration in intracranial interventions [21]. Techniques such as the two‐wire gooseneck snare bailout [22] and balloon‐assisted straightening maneuvers [18] reflect procedural creativity, yet still relied on fluoroscopic precision and specialized hardware.

**TABLE 1:** Comparative analysis of published foreign body retrieval cases involving fractured catheters, stents, or coils.

Study (Author, Year)	Setting	Device type	Fragment location	Retrieval access site	Retrieval method	Adjunct imaging/guidance	Special maneuvers	Complications	Outcome
Kadoya et al. [22]	High‐resource, Japan	Crosser catheter tip	Anterior tibial artery	Femoral artery	Gooseneck snare with two wires	Fluoroscopy	Two‐wire technique with balloon dilation	None	Successful
Xie et al. [16]	High‐resource, China	Central venous catheter fragment	Inferior vena cava	Right internal jugular vein	Gooseneck snare	Fluoroscopy	None reported	None	Successful
Rahimov et al. [18]	High‐resource, Azerbaijan	Judkins diagnostic catheter	Radial and brachial artery (kinked)	Right radial artery	Balloon‐assisted straightening	Fluoroscopy	Distal balloon inflation to anchor and pull	None	Successful
Miura et al. [15]	High‐resource, Japan	Coil	ICA (internal carotid artery)	Right femoral artery	Snare advanced over microcatheter as monorail guide	Fluoroscopy	Monorail snare adjustment in tortuous vessel	None	Successful
Antono et al. [17]	High‐resource, Indonesia	Double‐lumen catheter guidewire	Right atrium	Right internal jugular vein	Percutaneous loop‐wire snaring	Fluoroscopy	MemoPartTM snare under direct imaging	Minimal bleeding at access site	Successful
Yassin et al. [20]	High‐resource, Egypt	Unexpanded stent	LM ostium to aorta	Radial and femoral arteries	Dual guide with snare (ping‐pong technique)	Fluoroscopy	Stent fixation + traction with snare	LM stent deformation (corrected)	Successful
Chen et al. [19]	High‐resource, Taiwan	Angiocatheter	Brachial artery	Left radial artery	Snare under ultrasound guidance	Sonography	Real‐time ultrasound‐guided loop capture	None	Successful
Webb et al. [21]	High‐resource, USA	Microcatheter	Meningeal artery to carotid	Common carotid artery	Stent retriever with aspiration	Fluoroscopy	Multiple resheathing + aspiration	None	Successful

Unlike these reports, our technique required real‐time improvisation under fluoroscopic guidance using loop‐size optimization and sheath upsizing in the absence of advanced retrieval systems. Furthermore, we navigated severe vascular tortuosity, iliac dissection, and arrhythmogenic risk from an intracardiac foreign body—highlighting both the technical complexity and hemodynamic vulnerability of the patient. The successful outcome, with no surgical intervention and minimal complications, demonstrates the safety and replicability of snare‐based retrieval even in austere environments. This underscores the importance of disseminating simplified, cost‐effective retrieval algorithms that can be adapted in global health contexts where access to high‐end tools remains limited.

Collectively, these insights support the broader dissemination of simplified, cost‐effective retrieval protocols adaptable to constrained settings. Our experience underscores the feasibility of safe and effective catheter management in anatomically and logistically challenging cases, contributing to the evolving body of evidence guiding interventional cardiology in global health contexts.

## Author Contributions

**Abdul Qadir Nawabi:** formal analysis, project administration, supervision, validation, visualization, writing – original draft, writing – review and editing. **Christian Sanchez:** visualization, writing – original draft, writing – review and editing. **Aya Ziq:** conceptualization, writing – review and editing. **Mukhtaar Ahmed:** visualization, writing – review and editing. **Zarina Razaie:** data curation, resources, software, visualization, writing – review and editing. **Noor Ahmed Jamal:** supervision, writing – original draft, writing – review and editing. **Ahmad Jamshed Rezaie:** data curation, project administration, resources, supervision, validation, writing – review and editing.

## Ethics Statement

This manuscript does not include personal or medical details about any identifiable individual. Written consent was obtained from the patient as per the journal's patient consent guidelines.

## Consent

All patient data has been fully anonymized, and authors have obtained written informed consent to write about the case.

## Conflicts of Interest

The authors declare no conflicts of interest.

## Supporting information


**Figure S1:** ccr370785‐sup‐0001‐Figures.docx.

## Data Availability

Relevant data supporting the conclusions of this case report can be obtained from the corresponding author upon request.
