# Supplementary material for: Successful Retrieval of a Fractured Coronary Angiography Catheter Using Snare Technique: A First Case Report From Afghanistan
Source: Clin Case Rep. 2025 Aug 27;13(9):e70785. doi: 10.1002/ccr3.70785 (PMC12391723; doi:10.1002/ccr3.70785)
Supplement: Supplementary file 1 — Figure S1: ccr370785‐sup‐0001‐Figures.docx. [file CCR3-13-e70785-s001.docx]

**(FIGURE 1) Initial Imaging of Fractured Catheter**. Angiogram showing the fractured catheter segment lodged in the left external iliac artery, with guidewire and catheter pathway visible

**(FIGURE 2) Fractured Catheter Migration**. The catheter fragment is visualized within the right internal iliac artery, illustrating its migration and procedural complexity.

**(FIGURE 3) Catheter and Wire Orientation.** Imaging reveals the proximal catheter fragment embedded in the left ventricular apex, highlighting arrhythmia risk and retrieval difficulty.

**(FIGURE 4) Abdominal Aorta and Iliac Artery Branching.** Angiographic view of the abdominal aorta and iliac branches, contextualizing the catheter’s path relative to major vascular structures.

**(FIGURE 5) Snaring of Looped Catheter.** The looped catheter fragment is secured by a Goose neck snare within an 11F sheath, demonstrating minimally invasive retrieval.

**(FIGURE 6).** **Procedural flowchart**. Stepwise schematic of fractured coronary catheter retrieval using snare technique.

**(FIGURE 7) Retrieved Medical Equipment.** This final image shows the (1) distal part of fractured catheter, (2) distal end of proximal fractured catheter, (3) SNARE, (4) 11 French sheath, (5) proximal end of distal fractured catheter, and (6) proximal end of catheter previously in the LV.
